# Multi-Modal Point-of-Care Diagnostics for COVID-19 Based on Acoustics and Symptoms

**DOI:** 10.1109/JTEHM.2023.3250700

**Published:** 2023-03-08

**Authors:** Srikanth Raj Chetupalli, Prashant Krishnan, Neeraj Sharma, Ananya Muguli, Rohit Kumar, Viral Nanda, Lancelot Mark Pinto, Prasanta Kumar Ghosh, Sriram Ganapathy

**Affiliations:** LEAP LaboratoryDepartment of Electrical EngineeringIndian Institute of Science 29120 Bengaluru 560012 India; P. D. Hinduja National Hospital and Medical Research Center Mumbai 400016 India

**Keywords:** COVID-19 diagnostics, acoustic bio-markers, point-of-care testing, multi-modal classification

## Abstract

Background: The COVID-19 pandemic has highlighted the need to invent alternative respiratory health diagnosis methodologies which provide improvement with respect to time, cost, physical distancing and detection performance. In this context, identifying acoustic bio-markers of respiratory diseases has received renewed interest. Objective: In this paper, we aim to design COVID-19 diagnostics based on analyzing the acoustics and symptoms data. Towards this, the data is composed of cough, breathing, and speech signals, and health symptoms record, collected using a web-application over a period of twenty months. Methods: We investigate the use of time-frequency features for acoustic signals and binary features for encoding different health symptoms. We experiment with use of classifiers like logistic regression, support vector machines and long-short term memory (LSTM) network models on the acoustic data, while decision tree models are proposed for the symptoms data. Results: We show that a multi-modal integration of inference from different acoustic signal categories and symptoms achieves an area-under-curve (AUC) of 96.3%, a statistically significant improvement when compared against any individual modality ( $p < 0.05$). Experimentation with different feature representations suggests that the mel-spectrogram acoustic features performs relatively better across the three kinds of acoustic signals. Further, a score analysis with data recorded from newer SARS-CoV-2 variants highlights the generalization ability of the proposed diagnostic approach for COVID-19 detection. Conclusion: The proposed method shows a promising direction for COVID-19 detection using a multi-modal dataset, while generalizing to new COVID variants.

## I. Introduction

The highly contagious variant of the coronavirus family, SARS-CoV-2, has resulted in a significant health crisis [1]. The outbreak was termed as the coronavirus disease 2019 (or COVID-19) and declared a pandemic in March-2020 [1]. The pathogenesis of COVID-19 suggests that the infection triggers the SARS-CoV-2 virus to replicate and migrate down the respiratory tract, to the epithelial cells in the lungs [2]. The symptoms of COVID-19 include fever, common cold, cough, chest congestion, breathing difficulties, dyspnea, and loss of smell (and/or taste) [3]. Easy access to COVID-19 screening methodology can help to identify and isolate infected individuals, and control the spread [4].

### A. Current Tests and Limitations

The current gold-standard in COVID-19 diagnosis is the reverse transcription polymerase chain reaction (RT-PCR) assay [5]. However, this diagnosis methodology has four major limitations, namely, i) the high cost, ii) need for expert supervision, iii) longer turnaround time for results, and iv) lack of physical distancing during sample collection. A widely used alternative to RT-PCR testing is the rapid antigen testing (RAT) methodology [6]. The sensitivity, at the predefined specificity, is lower compared to the RT-PCR test [7]. While the CT imaging is also useful for COVID diagnosis [8], it requires expensive machinery, and exposes the body to harmful radiations. In summary, there is a need to invent alternative testing methodologies which provide improvement with respect to time, cost, physical distancing and detection performance [9].

### B. Acoustics for Respiratory Diagnostics

For the identification of respiratory disorders, listening to the acoustic signatures using a sound amplifier placed on the chest was formalized by Laennec [10] as early as 1819. Several studies have shown the presence of wheezing and crackling sounds to indicate severity of asthma and pulmonary fibrosis, respectively [11]. The cough engages high velocity airflow to clear the respiratory pathways from secretions such as mucus, and foreign particles [12]. Analysis of cough sound recordings has gained considerable interest [13]. Studies have shown effectiveness in the detection of pertussis [14], tuberculosis [15], pneumonia [16], wet versus dry cough [17], and asthma [18]. The pulmonary disorders can also limit vital lung capacity, thereby inhibiting efficient speaking [19].

### C. Prior Work

Recently, drawn by the need to control the spread of COVID-19, multiple respiratory acoustic datasets have been created by different research groups. These include the COVID-19 Sounds dataset [20] by University of Cambridge (UK), Buenos Aires COVID-19 Cough dataset [21], COUGHVID dataset [22] by EPFL University (Switzerland), COVID-19 Open Cough dataset [23] by Virufy (US), COVID-19 audio dataset by voca.ai (US) (used in [24]), and the COVID-19 Cough dataset [25] by MIT (US). Our group has also released an open-access COVID-19 audio and symptoms dataset, named as the Coswara COVID-19 dataset [26], while also organizing data challenges using the data collected [27], [28].

Several studies have attempted a binary classification task on these datasets to detect individuals with COVID-19 infection. These works explore acoustic feature representations such as mel-frequency cepstral coefficients (MFCCs) [29], mel-spectrogram [25], [30], scalograms [31], glottal flow dynamics [32], and classifier models such as deep learning based neural networks (convolutional neural networks (CNNs) [31], recurrent neural networks (RNNs) [33], CNN based feature embeddings with support vector machines (SVM) [29] and CNN based residual networks [25], [30]. The detection performance is quantified using the area under the receiver operating characteristics curve (AUC).

Focusing on cough sound samples, Brown et al. [29] report an AUC of 0.82. Further, in the first Diagnosis of COVID-19 using Acoustics (DiCOVA) Challenge [34], 29 teams report AUC between $0.55-0.87$ on cough sound samples taken from a subset of Coswara dataset. A few studies have also explored using breathing [28], [29], [30] and sustained phonation of vowel sounds [32], [35] for COVID-19 detection.

The multi-modal analysis of cough (intensity and count), heart rate, respiratory rate, and temperature signals has been suggested as an approach to monitor recovery [36]. Menni et. al. [37] report an AUC of 0.74 using the symptom data while Zaobi et. al. [38] report an AUC of 0.90 with a broader set of symptoms. Further, Han et al. [39] have explored using symptom and voice datasets, jointly.

### D. Contributions

This paper makes the following contributions.

1) *Multi-modal fusion*: We explore COVID-19 detection using breathing, cough, and speech sound recordings, independently. Further, we show that a multi-modal approach of combining symptom information with the acoustic based classifiers results in a significant detection performance of 0.96 AUC ( $p < 0.0001$).
2) *Features and classifiers*: We explore different acoustic feature representations namely, mel-spectrograms, mel-frequency spectral coefficients (MFCCs), and low level descriptors based on voicing, energy and harmonics. On the classification front, we explore logistic regression (LR), linear support vector machines (SVMs) and long short term memory (LSTM) models.
3) *Score distribution analysis*: We analyze the score distribution of the best classifier model using data collected from beyond the model development stages, containing data recorded during the surge of the Omicron variant in India. The score analysis suggests that the proposed approach is robust to presence of newer SARS-CoV-2 variants of concern.

### E. Clinical Impact

The outbreak of COVID-19, and the resulting breakdown of the healthcare services in several countries, has necessitated the design of accurate, cost-effective, scalable and remote screening methodologies. The current testing methodologies, approved in most parts of the world, involve either a visit to a centralized facility or require additional sophisticated components and chemical reagents for remote testing. Further, the cost of testing may be restrictive for wide-spread screening. In this context, our study is placed among those, which explore non-invasive data such as respiratory sound samples and symptoms for respiratory health screening, with a focus on COVID-19 detection. The study is designed in a crowd-sourced setting, where the data is captured using the individual’s smart phone and the diagnosis result is made available within a minute of data collection. As the data capture is performed through the user’s own device, the testing is remote, cost-effective and with a reduced risk of further spread.

The paper presents the details of the data collection and the analysis. The findings from our study demonstrate an approach to achieve practically viable COVID-19 detection performance, by combining classifiers based on acoustic data such as breathing, cough, and speech, along with the information derived from the health symptom data. We also illustrate that the results from the proposed work are superior to the baseline systems proposed by various other research groups. Thus, we hypothesize that the study presents a screening solution that is deployable at population scale for quick, inexpensive and remote testing of COVID-19. Even though the diagnostic performance may be inferior to the gold standard PCR testing, the ease of using the tool encourages more participation from the population. Further, the tool can function as a screening methodology, recommending a followup testing with PCR for a subset of the subjects.

## II. Materials

### A. Dataset

The dataset used in the study is a subset of the open-access Coswara dataset^1^ [26]. The data collection procedure was approved by the Institutional Human Ethics Committee, at the Indian Institute of Science, Bangalore. The data was collected in a crowd-sourced manner, through various collaborating hospitals and health centers. Our team prepared a web-link^2^ which was shared with the volunteering subjects. The inclusion criteria for participants consisted of the need to have access to a personal smartphone, access to the internet and ability to comprehend English or one of the 6 Indian languages in which the tool was released. Anyone below the age of 15 was excluded from the study, as the current study only targeted adult population.^1^https://github.com/iiscleap/Coswara-Data^2^https://coswara.iisc.ac.in

**FIGURE 1.**
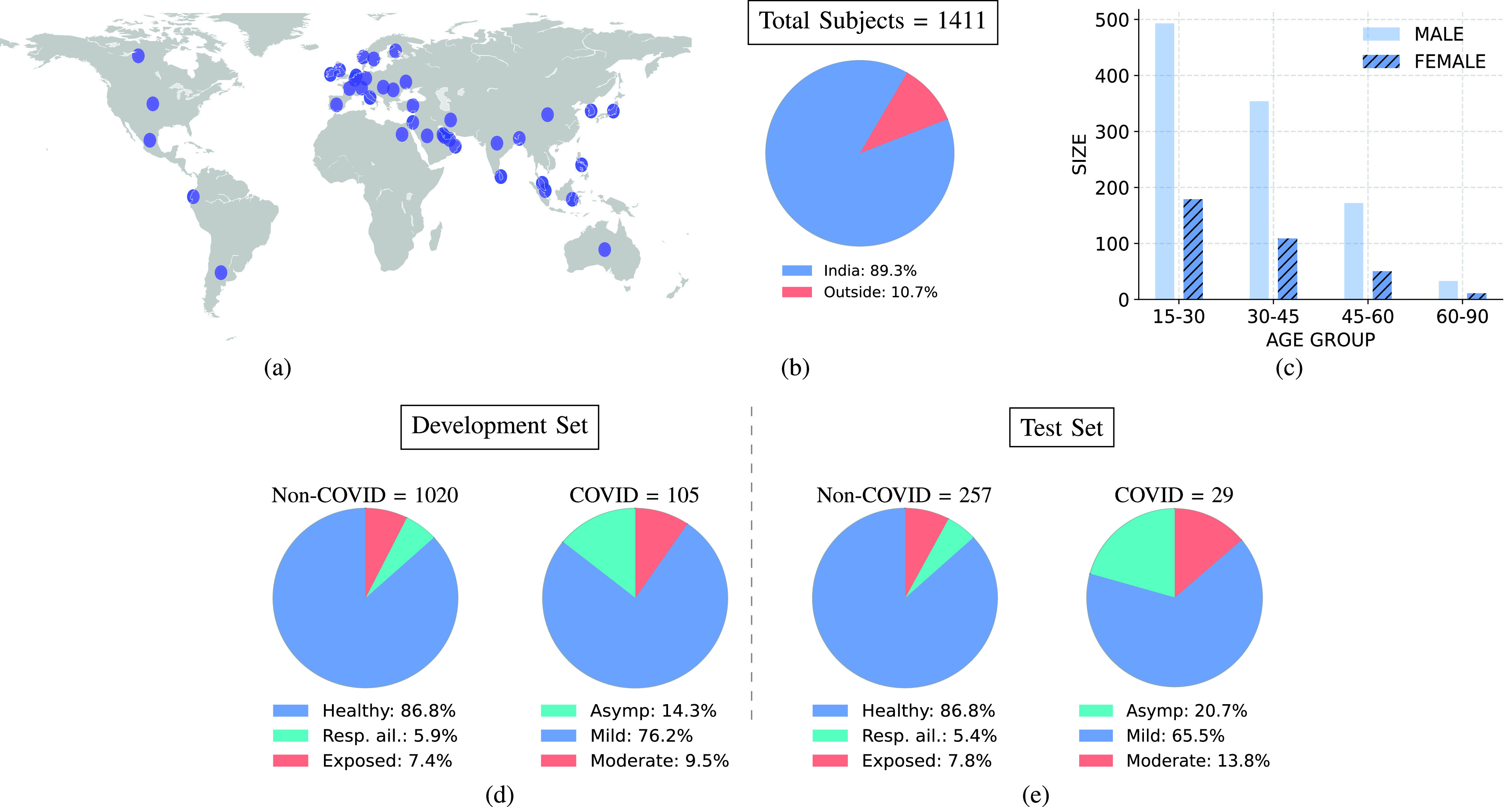
(a) The broad geographic distribution of subjects, (b) Percentage of subjects from India and outside, (c) age group and gender breakup. The distribution of sub-categories within COVID and non-COVID subjects in the (d) development set, and (e) test set.

We focus on the analysis of three sound categories, namely, (i) breathing-deep (or breathing), (ii) cough-heavy (or cough) and (iii) counting-normal (or speech), and the health symptoms data for the task of designing COVID-19 diagnostic solutions. An illustration of the geographic, age, and gender distribution of subjects is shown in Figure 1 (a-c). The subjects come from several countries, with 89.3% residing in India. A majority of the subjects fall in the $15-45$ age group, and are male (75.2%). We group the 1411 subjects into two pools. The first pool is referred to as *non-COVID* and comprises subjects which are either healthy, exposed to COVID-19 positive patients, or have pre-existing respiratory ailments. The second pool, referred to as *COVID*, comprises subjects who have mild, moderate, or asymptomatic COVID-19 infection. The health status of the subject corresponds to the self-reported health condition at the time of data collection (similar to other studies [29]). Majority of COVID-19 positive individuals and subjects with respiratory ailments came from hospitals collaborating in the data collection effort. For the positive subjects, the data was collected within $1-10$ days from the onset of the COVID-19 infection.

### B. Dataset Partitions

The subject pool of 1411 (135 COVID) subjects is divided into $80-20\%$ non-overlapping subject splits to obtain a development set and a test set, respectively, via stratified sampling. Both these sets contain data collected between April-2020 and May-2021.

#### 1) Development Data

The development set has 1125 (105 COVID-19 positive) subjects. The sub-category-wise distribution of the subjects is shown in Figure 1(d). We further divide the development set into training and validation folds using a five-fold cross-validation setup. The cross-validation data is used for hyper-parameter selection of the classifiers (shown in Figure 3 and described in Section IV).

**FIGURE 2.**
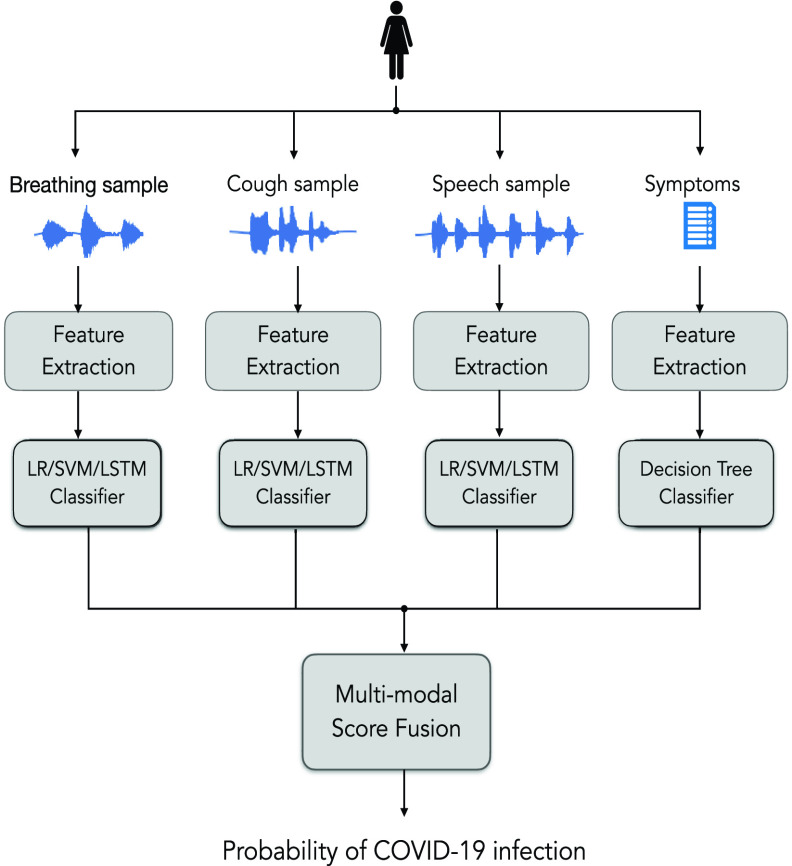
Schematic of the multi-modal approach for COVID-19 diagnostics.

**FIGURE 3.**
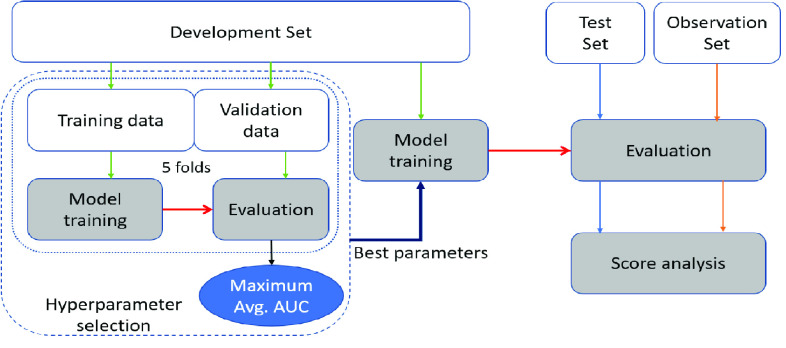
The dataset modeling and analysis. The development data is split into training and validation data, and used for five-fold validation experiments. The test set is used for evaluating the performance metrics. The observation set (described in Sec. IV-G) is used for score analysis.

#### 2) Test Data

The test set has 286 (29 COVID) subjects. The sub-category-wise distribution of the subjects is shown in Figure 1(e).

## III. Methods

### A. Acoustic Feature Representations

The Coswara data provides the sound samples as uncompressed WAV format audio files. We standardize all sound files to a sampling rate of 44.1kHz via re-sampling, and normalize the amplitude range of the audio samples (per-file) to ±1. This is followed by extraction of the following different kinds of spectro-temporal acoustic feature representations.

#### 1) Mel Spectrogram

A spectrogram is obtained by taking the (log) magnitude Fourier spectrum over short-time windows. A non-uniform frequency scale, namely the mel-scale, captures the non-uniform spectral energy distribution in audio signals [40]. We use 25 msec windows with a hop of 10 msec and 64 mel-filters. This results in a $64\times N_{k}$ dimensional feature matrix for the $k^{th}$ audio signal, where $N_{k}$ is the number of short-time segments.

#### 2) Mel-Frequency Cepstral Coefficients (MFCCs)

The MFCCs are a reduced dimensional representation obtained by applying the discrete cosine transform (DCT) to each column of the mel-spectrogram matrix and retaining only the top $M$ coefficients [40]. We choose $M$ as 40, resulting in a $40\times N_{k}$ dimensional feature matrix for the $k^{th}$ audio signal, where $N_{k}$ is the number of short-time segments.

#### 3) ComParE Low-Level Descriptors (LLDs)

This feature set was proposed in the INTERSPEECH 2013 Computational Paralinguistics Evaluation [41] and has been used in speech processing, music information retrieval, and sound analysis [42]. Here, each short-time audio segment (25 msec) is represented by a vector comprising of energy features (4 dimensional), voicing features (6 dimensional), and spectral features (55 dimensional). The constituents are described in Table 1. This feature set is a $65\times N_{k}$ dimensional feature.

**TABLE 1:** Description of Different Acoustic Features Used in Experiments With the LSTM Model

Acoustic Feature	Dimension	Description
Energy	4	Sum of auditory spectrum Sum of RASTA-style auditory spectrum RMS energy Zero-crossing rate
Voicing	6	Fundamental frequency Probability of voicing Log. harmonic-to-noise ratio Jitter (local, delta) Shimmer
Spectral	55	RASTA-style auditory spectrum 1-26 MFCC 1-14 Spectral Energy 250–650 Hz, 1 k-4 kHz Spectral roll-off point 0.25, 0.50, 0.90 Spectral flux, centroid, entropy, slope Spectral sharpness, harmonicity Spectral variance, skewness, kurtosis
ComParE LLDs	65	Energy + Voicing + Spectral
MFCC	40	MFCC 0–40
logmelspec	64	64 channel mel-scale spectrogram (in dB)

#### 4) Compare Functionals

Further studies have proposed statistical quantification of the temporal variability in the ComParE LLDs over the total audio signal duration [41]. The resulting feature set is referred to as the ComParE functionals. This feature set includes the inter-quartile ranges, mean, standard deviation, skewness, kurtosis, maximum, minimum, linear regression coefficients, and other statistics [42]. Altogether, these capture the temporal dynamics of the LLDs and represent it as a fixed length feature vector of 6373 dimensions. While all previous features are frame-level features, these are file-level features.

The same set of features are derived for the three sound categories, that is, breathing, cough and speech. Further, we also append the successive frame-wise derivatives and double derivatives to the spectrogram, MFCC and LLD feature sets, respectively [40]. This allows modeling the temporal variability in the features.

### B. Symptoms Feature Representation

We represent the health symptoms of each subject using a binary feature vector. The presence (or absence) of each of the symptoms, namely, cough, cold, fever, loss of smell, sore throat, diarrhea, fatigue and muscle pain, is encoded as a one (or zero) in an 8 dimensional vector.

### C. Classification Models

#### 1) For Acoustic Features

We explore two kinds of classifiers, namely, linear and non-linear classifiers. In the linear classifiers, we consider logistic regression and linear support vector machines (SVM). In the non-linear classifiers, we consider the bi-directional long short term memory (BLSTM) neural network with inputs being the frame-level features.

For the BLSTM, the input is fed to a stack of $L$ BLSTM layers with $h_{l}$ units followed by a pooling layer. The pooling layer performs averaging along the time dimension to generate a sequence level embedding for the input segment. The embedding is then fed to a linear layer with $h_{p}$ units followed by a *tanh* non-linearity. The output is then projected to scalar followed by a sigmoid activation that denotes the COVID probability.

For the frame-level classifiers (LR/SVM), the classifier is trained to predict the decision at each frame and a file-level score is obtained by averaging the individual frame-level scores. For the segment-level LSTM classifier, we sample segments of size 0.5 s with 0.1 s hop size, and the classifier is trained to predict segment-level scores. The average of the scores for all the segments is used as the file-level score.

#### 2) For Symptoms Features

We consider a decision-tree classifier on the symptoms features. Each node in the tree is associated with a “binary-test” on the value of a feature dimension and the edges drawn out of a node correspond to the two possible outcomes of the test. The leaf nodes are associated with a posterior probability distribution over the classes. The Gini criterion is used [43] to find the optimal tree structure.

### D. Dealing With Class Imbalance

The classifier models are trained using a *balanced loss* configuration [44]. Let, $N_{c}$ and $N_{nc}$ be the count of COVID and non-COVID subjects used in training, respectively. Let $r=N_{c}/N_{nc}$ be the class ratio. Then, the total loss is,

\begin{equation*} L = \sum _{x \in c} l(x) + r \sum _{x \in nc} l(x) \tag{1}\end{equation*}

where, $x$ denotes the input sample, and $c$ ( $nc$) denotes the set of COVID (non-COVID) samples.

### E. Performance Metrics

We use the area-under-the curve (AUC) measure of the receiver operating characteristic curve (ROC) [45] as the primary performance metric. Let $\hat {N}_{c}$ and $\hat {N}_{nc}$ denote the count of correctly predicted COVID and non-COVID subjects, respectively. Then we have,

\begin{equation*} \text {sensitivity}= \frac {\hat {N}_{c}}{N_{c}}, \text {specificity}= \frac {\hat {N}_{nc}}{N_{nc}} \tag{2}\end{equation*}

We compute the ROC curve by varying the decision threshold from 0 to 1 in steps of 10^−4^ and obtaining the specificity (and sensitivity) at each of these thresholds. The AUC is computed using the trapezoidal rule [46]. The positive predictive value (PPV) and the negative predictive value (NPV) is,

\begin{equation*} \text {PPV}= \frac {\hat {N}_{c}}{N_{c} + (N_{nc}-\hat {N}_{nc})}, \text {NPV}= \frac {\hat {N}_{nc}}{N_{nc} + (N_{c}-\hat {N}_{c})} \tag{3}\end{equation*}

### F. Multi-Modal Fusion

The block schematic of the multi-modal diagnostic tool proposed in this work is shown in Figure 2. We explore the fusion of predicted probability scores from the different categories of acoustic data (cough, breathing and speech). Further, we also explore a multi-modal approach in which the scores from acoustic data are combined with that from symptoms data. We use score averaging as the fusion scheme and the final predicted score using the four modalities is computed as,

\begin{equation*} p = \left ({p_{\textrm {cough}} + p_{\textrm {breathing}} + p_{\textrm {speech}} + p_{\textrm {symptoms}} }\right)/4, \tag{4}\end{equation*}

where $p_{m}$ is the prediction score obtained for the modality $m$.

### G. Implementation

The acoustic feature extraction pipelines are implemented using the Librosa [47], Torch-audio [48], and OpenSmile [49] Python packages. The LR and SVM classifiers are implemented using the Scikit-learn package [46] and the LSTM is implemented^3^ using the Pytorch package [48].^3^The code scripts used in this study is available at: https://github. com/iiscleap/MuDiCov

## IV. Experiments and Results

The training, validation, and evaluation setup is illustrated in Figure 3. A five-fold validation is used to select the hyper-parameters, namely, $\lambda $ for LR and SVM models, and minimum number of samples in leaf nodes for the decision-tree classifier. For LSTM, the number of hidden units $h_{l}$, linear projection dimension $h_{p}$, number of layers $L$ and the LSTM cell type constitute the hyper-parameter set. The hyper-parameter setting corresponding to the best average AUC measure over the five-folds is finally selected. These values are provided in Table 3. Subsequently, the classifier, with the selected hyper-parameter value, is trained on the entire development set, and evaluated on the test set.

**TABLE 2:** Area Under the ROC Curve (AUC) Performance Obtained With Different Feature and Classifier Combinations (Along With the 95% Confidence Interval in Five-Fold Validation.)

Feature Name	Feature Type	Feature Dimension	Classifier	Avg. VAL. AUC			Test AUC		
				Breathing	Cough	Speech	Breathing	Cough	Speech
ComParE Functionals	File level	$6373\times 1$	LR	0.75(± 0.02)	0.65(± 0.04)	0.72(± 0.05)	0.78	0.74	0.79
			Lin-SVM	0.75(± 0.01)	0.64(± 0.04)	0.71(± 0.06)	0.75	0.74	0.79
			RBF-SVM	0.73(± 0.06)	0.64(± 0.06)	0.67(± 0.09)	0.79	0.74	0.73
ComParE LLDs	Frame level	$195\times 1$	LR	0.64(± 0.10)	0.63(± 0.03)	0.61(± 0.09)	0.63	0.65	0.62
			Lin-SVM	0.62(± 0.04)	0.51(± 0.06)	0.54(± 0.09)	0.60	0.61	0.65
			LSTM	0.72(± 0.13)	0.58(± 0.12)	0.69(± 0.09)	0.72	0.72	0.76
logMelspec	Frame level	$192\times 1$	LR	0.61(± 0.10)	0.65(± 0.08)	0.59(± 0.10)	0.65	0.65	0.63
			Lin-SVM	0.62(± 0.07)	0.62(± 0.06)	0.59(± 0.11)	0.66	0.67	0.60
			LSTM	0.75(± 0.06)	0.64(± 0.05)	0.73(± 0.07)	0.81	0.85	0.80
MFCC	Frame level	$120\times 1$	LR	0.61(± 0.08)	0.64(± 0.07)	0.60(± 0.10)	0.67	0.67	0.63
			Lin-SVM	0.62(± 0.07)	0.63(± 0.04)	0.57(± 0.10)	0.70	0.66	0.53
			LSTM	0.74(± 0.12)	0.61(± 0.07)	0.70(± 0.10)	0.74	0.63	0.76

**TABLE 3:** Hyperparameters Found From Validation Experiments. $\ell_{2}$ Regularizer ( $\lambda$) for LR/SVM Models. The LSTM Model Has $L$ LSTM Layers With $h_{l}$ Units and a Linear Layer With $h_{p}$ Units

Sound Category	Classifier	$\lambda $	$L, h_{l}, h_{p}$
Breathing	LR	$1e^{-4}$	–
	Lin. SVM	$1e^{-4}$	–
	RBF SVM	10	–
	LSTM	–	1, 64, 256
Cough	LR	$1e^{-3}$	–
	Lin. SVM	$1e^{-4}$	–
	RBF SVM	1	–
	LSTM	–	1, 128, 256
Speech	LR	$1e^{-4}$	–
	Lin. SVM	$1e^{-5}$	–
	RBF SVM	10	–
	LSTM	–	3, 128, 128

### A. Acoustic Classifiers

The performance of the three classifiers, namely, LR, Lin-SVM, and LSTM, on different acoustic feature sets extracted from each sound category are reported in Table 2.

#### 1) With Frame-Level Features

With mel-spectrogram features, the average validation AUC ranges from $0.54-0.75$ across the different classifier models. The LSTM model outperforms the LR and SVM models for all three sound categories. On the test set, the LSTM model gives an AUC in the range of $0.81-0.85$. With MFCC features, for all models, the average validation performance is lower than (or similar to) that obtained with mel-spectrogram features. With ComParE LLDs features, the average validation AUC ranges from $0.51-0.72$. In summary, across the three sound categories, the LSTM model gives better performance for the majority of the features explored in this work.

#### 2) With File-Level Features

We train and evaluate the performance of linear classifiers with the ComParE Functionals (see Table 2). The performance is consistently better for the breathing sound category. The test set performance ranges from $0.73-0.79$ AUC. Both LR and SVM gave a similar performance. The validation performance of the ComParE functionals is comparable to the LSTM classifier trained on mel-spectrogram features, however for the test set, the performance of LSTM classifier trained on mel-spectrogram features (frame-level) is better across the three sound categories.

### B. Symptom Classifier

Let $N_{c,s}$ and $N_{c,ws}$ denote the count of COVID subjects with and without symptom $s$, respectively. Similarly, $N_{nc,s}$ and $N_{nc,ws}$ denote the count of non-COVID subjects with and without symptom $s$, respectively. Then, the odds ratio $r_{s}$ is defined as,

\begin{equation*} \color {Black}{ r_{s} = \frac {N_{c,s}/N_{nc,s}}{N_{c,ws}/N_{nc,ws}}} \tag{5}\end{equation*}

**FIGURE 4.**
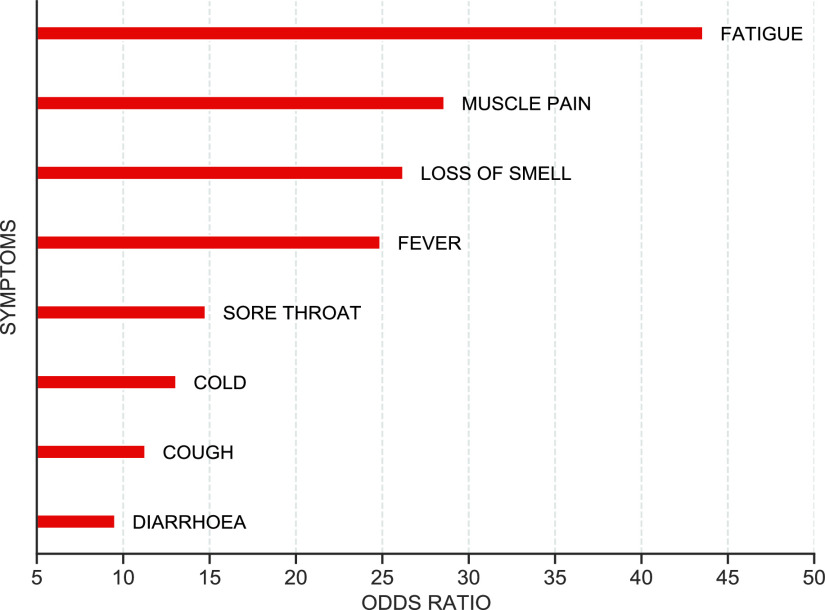
Odds ratio of the symptoms data in the Coswara dataset.

Figure 4 depicts the odds ratio computed from the training set for each of the eight symptoms. The odds ratio is higher for fatigue, muscle pain, and loss of smell.

**FIGURE 5.**
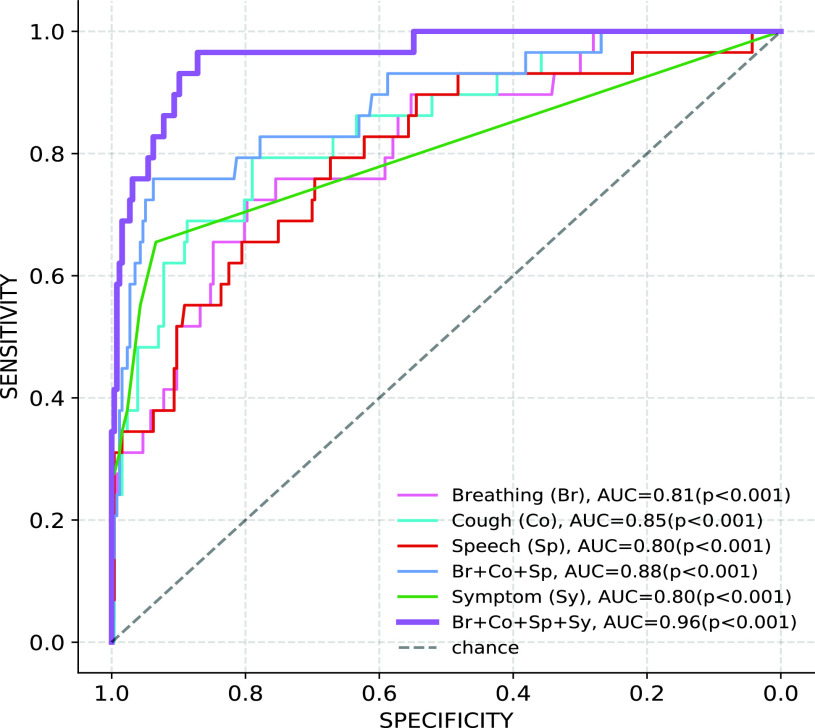
Test ROCs of the individual and the fusion systems for the LSTM classifier with mel-spectrogram features. The AUC significance was computed using the Mann Whitney statistical test [50].

Figure 8 shows the decision tree classifier trained using the symptoms features. The hyper-parameter, minimum number of leaf-nodes, is chosen using cross-validation, which is found to be 25. The isolated symptoms of loss of smell and fatigue are assigned probability greater than 0.9 (higher odds ratio seen in Figure 4). The symptom of sore throat has the smallest probability of 0.764. Overall, the model achieves a test AUC of 0.80 (Figure 5).

**FIGURE 6.**
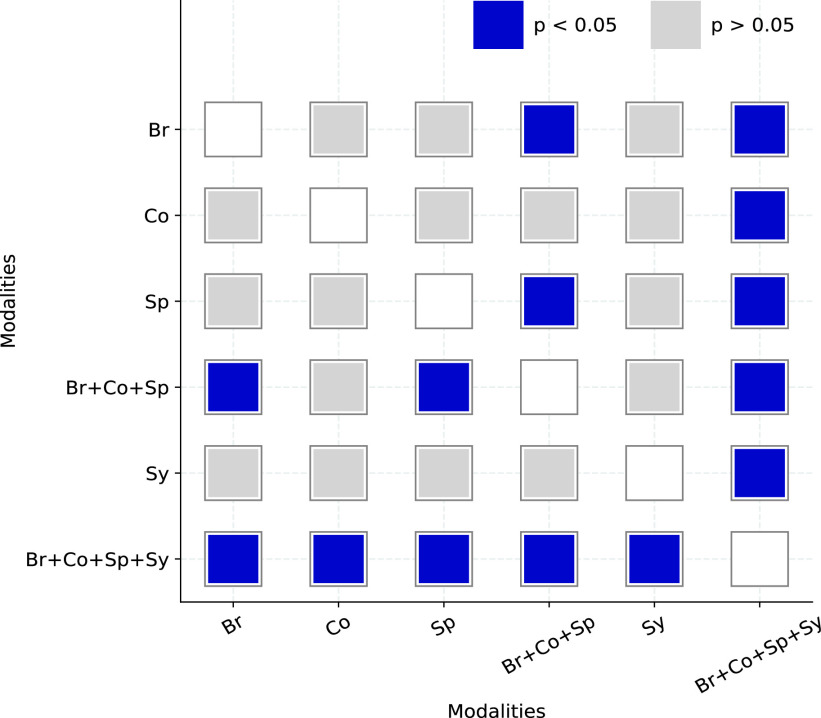
The DeLong [51] ignificance testing between pairs of ROCs. The significant ( $p < 0.05$) comparisons are highlighted.

**FIGURE 7.**
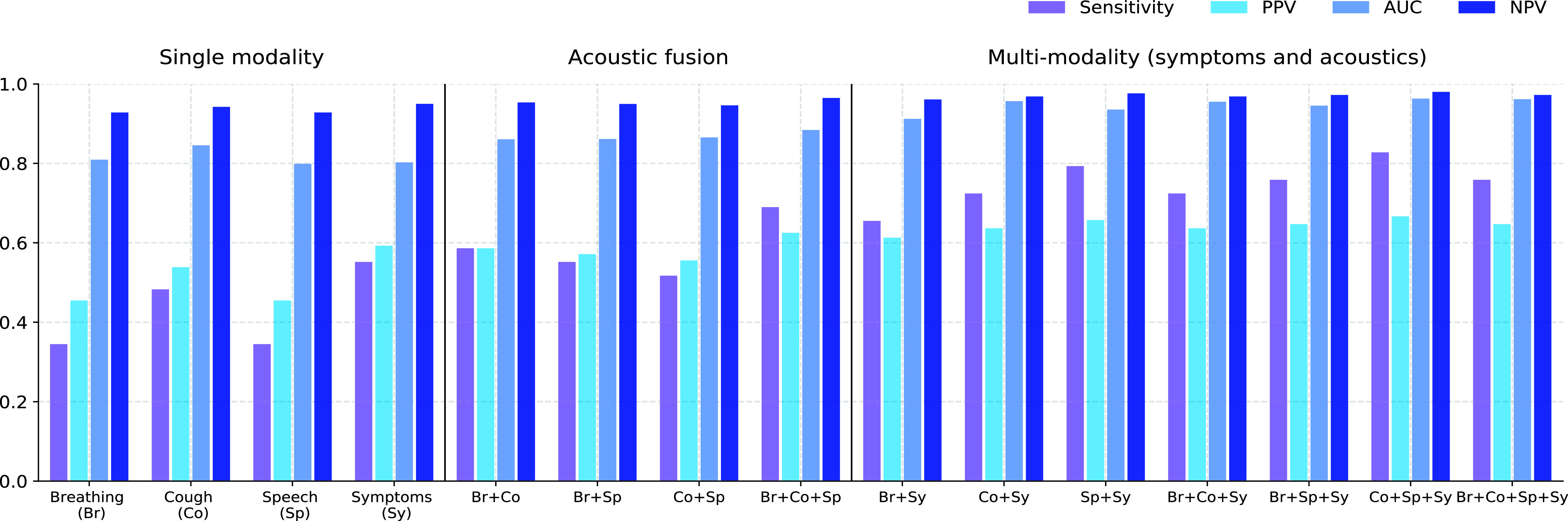
Performance of the individual modalities and score fusion of multiple modalities. Here the sensitivity, Positive Predictive Value (PPV) and Negative Predictive Value (NPV) are measured at a specificity of 95%.

**FIGURE 8.**
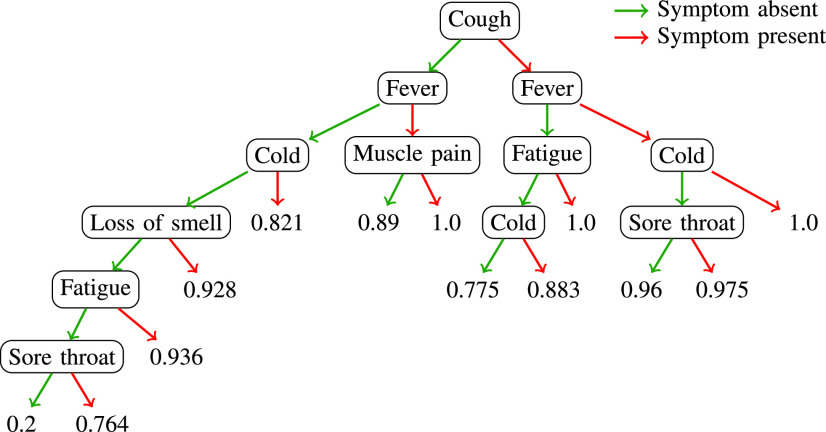
Decision tree model trained on the symptoms data. The value at the leaf node is the probability score for the COVID class.

### C. Multi-Modal Fusion

We explore the possibility of fusing predictions obtained from models associated with different acoustic categories (LSTM classifier with mel spectrogram features) and symptoms. Table 4 depicts the cross-correlation coefficients between pairs of predicted test set scores, obtained from different data modalities. The correlation coefficient is less than 0.5 for all the pairs of modalities. The scores predicted using symptoms have less correlation with scores from all the sound categories. The low cross-correlation suggests that score fusion across the categories can further improve the classification performance. Figure 5 shows the test ROCs for the individual modalities, fusion of the acoustic categories, and the fusion of all the four categories. The fusion of the three acoustic categories yields an improvement over all the individual categories, and achieves an AUC of 0.88. The multi-modal fusion of the four categories further improves the overall AUC to 0.96, a significant improvement over the ROC-AUC performance of individual modalities. We have also reported the p-value computed using the Mann-Whitney test [50] (see Figure 5 legend). For all the ROC curves, the AUC values are found to be statistically significant.

**TABLE 4:** Crosscorrelation Coefficient Between Sets of Test Scores Obtained From Sound Category Specific Classifiers

Category	Breathing	Cough	Speech	Symptom
Breathing	1.0	0.497	0.421	0.038
Cough	0.497	1.0	0.460	0.031
Speech	0.421	0.460	1.0	0.040
Symptom	0.038	0.031	0.040	1.0

We have also performed a pair-wise comparison of the ROCs using the DeLong statistical test [51] to compute the significance value for the observed difference in AUCs across modalities. This is shown in Figure 6. The difference between the pairs of single acoustic categories are not found to be significant. The ROC obtained with a fusion of all three sound categories (Br+Co+Sp) is found to be significantly different ( $p < 0.05$) from that of breathing and speech modality. Further, the ROC obtained using a multi-modal fusion of acoustics and symptoms (Br+Co+Sp+Sy) is found to be significantly different ( $p < 0.01$) from all other ROCs.

Figure 7 shows the sensitivity, positive predictive value (PPV), and negative predictive value (NPV) measured at a specificity of 95%, and test AUC for different modalities. The fusion of the three acoustic categories is found to improve the test AUC by 3% points over the best performing individual sound category. Figure 7 also shows the performance for fusion of symptoms and the acoustic modalities as well as the fusion of pairs of acoustic categories. We see that fusion with symptoms improves the performance of all the acoustic based classifiers. The fusion of all the four modalities achieves the test AUC of 0.96, an absolute improvement of 8% compared to the fusion of the acoustic categories alone. At 95% specificity, a sensitivity of 76% is achieved for the fusion of all modalities. The corresponding PPV is 0.65, with a NPV value of 0.97. At the operating point of 90% specificity, the sensitivity improves to 89.7% (false negative rate of 10.3%).

**FIGURE 9.**
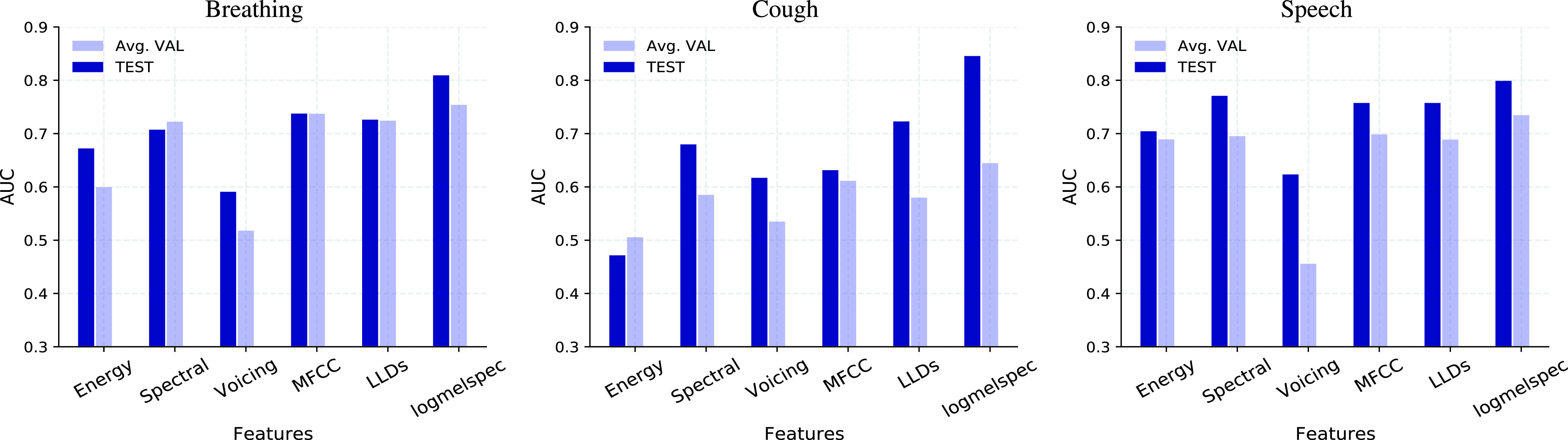
Performance of the acoustic fusion LSTM classifier trained with different features. A description of these features is provided in Table 1.

Using the LSTM model, we also analyzed the variability in the AUC performance obtained with different subsets of acoustic features. These included the energy, spectral, and voicing features extracted from the acoustic signals (see Table 1). The resulting average validation and test set performance is shown in Figure 9. For breathing modality, voicing features performed poorer than all other feature sets. The spectral features performed similar to MFCCs and LLDs. For cough modality, the energy features performed poorer than all other features. Here again, the spectral features performed similar to MFCCs and LLDs. For speech modality, the energy features performed similar to spectral features.

### D. Impact of Demographics

In the dataset, a majority of subjects (89.3%) resided in India. As India is a country with many spoken languages, we analyzed the impact of language/dialectal variations on the prediction scores. For this analysis, we divided the test set from India into two groups, namely, (a) the subjects coming from Southern India (SI) who belonged to non-Hindi speaking region, and (b) subjects coming from the rest of India (RI) with Hindi as their native language. For each sound category, we compared the COVID/non-COVID population score distribution obtained from the SI and RI partitions using the Mann Whitney statistical test [50]. The difference was not found to be significant ( $p>0.1$), suggesting that there was no impact of language/dialectal variations within India.

### E. Bias Analysis - Age, Gender, and Comorbidity

To understand how factors such as gender, age and comorbidity impact the COVID-19 score prediction, we carried out additional analysis. We focused on comparing the distributional similarity of the predicted COVID-19 score for different sub-populations of subjects in the test set. The sub-populations were created by grouping together subjects based on comorbidity (presence or absence), gender (male or female), and age (< or $\ge40$ years). Using the collected health data, a subject with diabetes, hypertension, ischemic heart disease, or any other pre-existing ailments was considered to have a comorbidity. The Mann-Whitney U test [50] was used to statistically compare the COVID-19 score distributions of sub-population with the COVID–19 scores for the full test set subject population. The results showed no significant impact of age and comorbidity. A significant bias based on gender was found in this analysis. A summary of p-values obtained from the statistical test is provided in Table 5. To overcome the gender bias in the results, we experimented with balancing the gender ratio in the training data by oversampling and re-trained the acoustic classifier models. This system, referred to as balanced in Table 5, did not contain significant bias related to the factors of gender, age, or comorbidity. Further, the gender balancing of the training data achieved the same overall AUC results of the original system.

**TABLE 5:** Bias Analysis by Comparing the Probability Distributions of Population Subgroups. $Orig.$ Refers to the Original Model (Fusion), and $Bal.$ Refers to the Model Trained With Gender Balancing. Here, $s.$ Corresponds to Statistically Significant Bias, While $n.s.$ Refers to Non-Significant Bias

Factors	p-value		
	Orig.	Bal.
Gender (Male vs Female)	p=0.01 (s.)	p=0.21 (n.s.)
Age (< 40 vs >=40)	p=0.19 (n.s.)	p=0.12 (n.s.)
Comorbidity (Present vs Absent)	p=0.39 (n.s.)	p=0.45 (n.s.)

### F. Comparison With Prior Work

We compared the performance of the proposed multi-modal approach with (i, ii) the approaches in [29] and [30] which use the breathing and cough modalities and (iii) the approach in [39] using speech and symptoms. We implemented the classification models used in [29], [30], and [39], and evaluated the performance on the dataset used in our present study. For the works by Brown et. al. [29], and Coppock et. al. [30] we used the codes made available by the authors.^4^, ^5^ Table 6 reports these results. The performance of the approaches in [29] and [30] is poor compared to the proposed approach. However, the approach of [39], which uses a 384 dimensional subset of the ComParE functional features, is comparable to the current work for the speech category. The performance of the SVM classifier for the symptom features is better than the decision tree classifier. But the performance of the score fusion system is found to be inferior to the proposed approach.^4^https://github.com/cam-mobsys/covid19-sounds-kdd20^5^https://github.com/glam-imperial/CIdeR

**TABLE 6:** Comparison of AUCs % Obtained on Test Set Using Methods in Prior Works and Method Proposed in Our Work

Method	Cough	Breath.	Speech	Sym.	All
Coppock et. al. [30]	0.75	0.70	–	–	0.77
Brown et. al. [29]	0.63	0.75	–	–	0.73
Han et. al. [39]	–	–	0.71	0.82	0.85
Proposed	0.81	0.85	0.80	0.80	0.96

### G. Generalizability Analysis

To understand the generalizability of the developed model to data collected subsequent to the model development, we analyzed additional data collected from May-2021 to Feb-2021 in the Coswara dataset. During this timeline there was a spread of newer SARS-CoV-2 variants, such as Delta and Omicron.

#### 1) Observation Set-1

**FIGURE 10.**
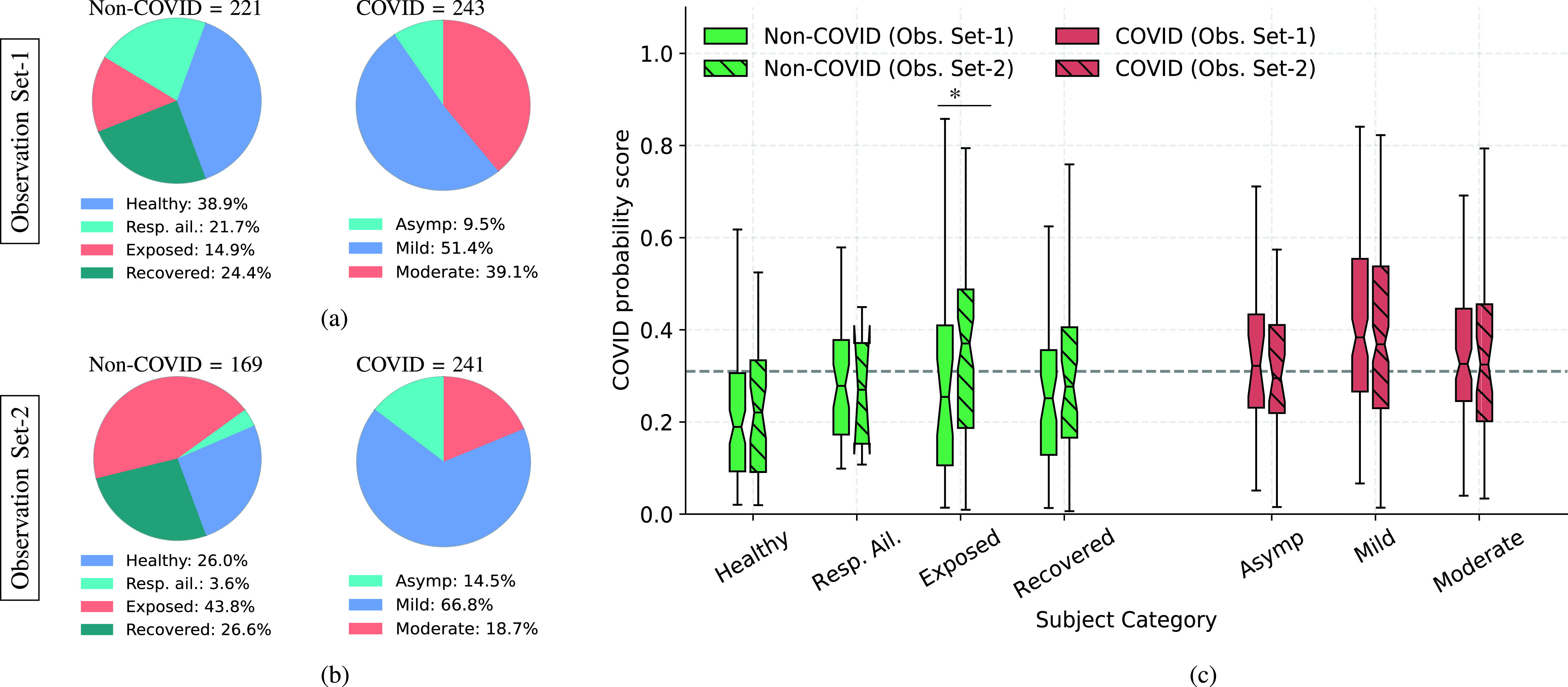
(a,b) Two different observation sets collected between 08-May-2021 and 30-Nov-2021 and between 01-Dec-2021 and 28-Feb-2022, respectively, (c) A box plot illustration of COVID probability score distribution for the samples in the two different observation sets. The COVID probability scores are obtained using the acoustic fusion LSTM classifier model. For each subject category, we did a Mann Whitney statistical test between scores for participants from Obs. Set-1 and that from Obs. Set-2. None of the pairs, except the exposed category, showed statistical significance ( $p < 0.05$) between the score distributions from the two observation sets. The horizontal dashed line indicates the decision threshold for 95% specificity on the test set.

The Observation Set-1 is contains data collected from subjects between 08-May-2021 and 30-Nov-2021 as well as the data from recovered subjects. There are a total of 464 (243 COVID) subjects. The category-wise distribution of subjects is shown in Figure 10(a).

#### 2) Observation Set-2

The Observation Set-2 data, collected between 01-Dec-2021 and 28-Feb-2022, contains data from 410 (241 COVID) subjects. This data was collected during the surge of the SARS-Cov-2 Omicron variant in India [52]. The category-wise distribution of subjects is shown in Figure 10(b). Thus, this set provides a platform for testing the generalizability of the models to newer variants.

We use the acoustic fusion of the LSTM classifiers with the symptom classifier for the analysis on the observation sets. The performance results on the two observation sets are reported in Table 7. As seen in this table, the AUC results generalize well to these observation sets, even though the model was trained on data prior to this data collection period and the COVID prevalence is different. We analyze the score distributions (Figure 10(c)). Each (vertical) box represents 25% (lower edge) and 75% percentile (upper edge) cut-offs, and the notch represents the median value. The two whiskers correspond to the minimum and maximum scores after outlier rejection. We also depict the operating threshold corresponding to the 95% specificity operating point. The distributions corresponding to the non-COVID subject category are further broken down into healthy, respiratory ailments (Resp. Ail), exposed, and recovered sub-categories. The distribution corresponding to the COVID category is broken down into asymptomatic, mild and moderate subject sub-categories. The score distribution for the healthy subjects is well below the threshold. The scores of the subjects with pre-existing respiratory ailments shows an upward trend, indicating the likelihood of more false-alarms for such subjects. The score distribution of the subjects who are exposed to COVID patients in observation set-II shows a higher median shift indicating that many of the participants may have been infected, although they were not diagnosed at the time of data collection. A larger spread in range of score is also observed for the subjects who have recovered from COVID (at least 10 days after the onset of the infection), indicating that the respiratory system may not have completely returned to the healthy state.

**TABLE 7:** Performance on the Observation Set-I, Set-II. The Senstivity, PPV and NPV Values are Measured at 95% Specificity

Comparison	AUC	Sensitivity	PPV	NPV
non-COVID healthy				
vs Mild	0.87, 0.86	0.30, 0.47	0.90, 0.97	0.49, 0.34
vs Moderate	0.86, 0.84	0.22, 0.40	0.84, 0.90	0.53, 0.61
vs Asymp.	0.77, 0.67	0.09, 0.14	0.33, 0.71	0.80, 0.59
vs All	0.85, 0.83	0.25, 0.41	0.94, 0.98	0.31, 0.23

For the asymptomatic COVID subjects, more than 50% of the asymptomatic subjects are correctly classified by the fusion system. Further, the score distributions obtained for Observation Set-1 (pre-Omicron) and Observation Set-2 (Omicron) had no statistically significant difference, except for the exposed condition. This suggests that the model may be robust to the newer variants of the SARS-Cov-2 variants. A recent work also explored the possibility of detecting the variants of COVID-19 from audio data [53].

## V. Discussion

Comparison With Prior Studies [29], [30], [39]: Many of the past studies were relatively small scale studies (62 COVID positive subjects in [30], 141 positive subjects in [39] and 235 COVID positive subjects in [30]), while our study involved 625 COVID-19 positive subjects. The works reported in [29], [30] had collected only two modalities of audio, namely cough and breathing. In these studies, there was no validation of the COVID positive labels as they were collected in a truly crowd-sourced manner. The best models achieved an AUC of 0.79 [39], 0.80 [29] and 0.84 [30] in these studies.

In our proposed study, 9 variants of audio-based data are collected, including 2 types of cough, 2 types of breathing, 3 vowel sounds and 2 types of counting speech. The study also collected a rich set of meta-data including pre-existing conditions, comorbidity, current symptoms, vaccination status and demographic information. The data from COVID-19 positive subjects and a subset of the non-COVID subjects came from hospitals and healthcare centers, where the positive status was ascertained with an RT-PCR test. Our proposed study details various feature and classifier choices to identify the best set of features, models and parameters. A large held-out observation set is used for score analysis which reflects novel data recorded after the analysis. In these observations sets, the proposed models are seen to generalize well and also generate score distributions that interpretable. More efforts in improving the interpretability of COVID-19 detection using audio can be found in [54] and [55].

In contrast with prior works, our work is the first of its kind to analyze the model performance on subjects who are exposed to COVID-19 (but not tested positive), subjects with pre-existing respiratory ailments, subjects who had recovered from COVID-19 and differentiate this with asymptomatic/symptomatic COVID-19 subjects (Figure 10). Furthermore, all the data used and models developed have been released as open-source, which was not the case in many of the previous studies.

## VI. Conclusion

We proposed, designed and evaluated a COVID-19 diagnostic approach based on using multi-modal data of acoustics and symptoms. The presented study used data from the Coswara dataset, an open-access dataset. This dataset contains sound samples and symptoms data collected from human subjects, with and without COVID-19 infection. We explored the use of different modalities, namely, breathing, cough, speech, and symptoms for COVID-19 prediction. This included experimentation with different kinds of acoustic feature representations, and classifier models. It was found that the LSTM model, operating at frame-level, trained with mel-spectrogram acoustic features outperformed other model and feature combinations. Further, we found that simple prediction score averaging to fuse information obtained from models trained on individual modalities significantly outperformed the rest. The fusion system achieves 76% sensitivity at 95% specificity. We also analyzed the score distribution obtained on recently collected data, associated with newer SARS-CoV-2 variants causing COVID-19. The analysis highlighted the robustness of the proposed approach. In summary, the paper proposes a methodology for rapid, cost-effective, and scalable screening tool for COVID-19.
